# Adding left atrial appendage closure to open heart surgery provides protection from ischemic brain injury six years after surgery independently of atrial fibrillation history: the LAACS randomized study

**DOI:** 10.1186/s13019-018-0740-7

**Published:** 2018-05-23

**Authors:** Jesper Park-Hansen, Susanne J.V. Holme, Akhmadjon Irmukhamedov, Christian L. Carranza, Anders M. Greve, Gina Al-Farra, Robert G. C. Riis, Brian Nilsson, Johan S.R. Clausen, Anne S. Nørskov, Christina R. Kruuse, Egill Rostrup, Helena Dominguez

**Affiliations:** 1 Department of Cardiology, Bispebjerg and Frederiksberg University Hospital, Nordre Fasanvej 57, DK-2000 Frederiksberg, Denmark; 2 Department of Biomedicine, University of Copenhagen, Copenhagen, Denmark; 3 Department of Thoracic Surgery, Rigshospitalet, Copenhagen, Denmark; 4 Department of Thoracic Surgery, Odense University Hospital, Odense, Denmark; 5 Department of Radiology, Herlev Gentofte University Hospital, Herlev, Denmark; 6 Department of Radiology, Bispebjerg and Frederiksberg Hospital, Frederiksberg, Denmark; 7 Department of Cardiology, Hvidovre University Hospital, Copenhagen, Denmark; 8 Department Neurology, Neurovascular Research Unit, Herlev Gentofte Hospital, Herlev, Denmark; 9 Mental Health Center Glostrup, Copenhagen, Denmark

**Keywords:** Atrial fibrillation, Heart surgery, Left atrial appendage closure, Stroke

## Abstract

**Background:**

Open heart surgery is associated with high occurrence of atrial fibrillation (AF), subsequently increasing the risk of post-operative ischemic stroke. Concomitant with open heart surgery, a cardiac ablation procedure is commonly performed in patients with known AF, often followed by left atrial appendage closure with surgery (LAACS). However, the protective effect of LAACS on the risk of cerebral ischemia following cardiac surgery remains controversial. We have studied whether LAACS in addition to open heart surgery protects against post-operative ischemic brain injury regardless of a previous AF diagnosis.

**Methods:**

One hundred eighty-seven patients scheduled for open heart surgery were enrolled in a prospective, open-label clinical trial and randomized to concomitant LAACS vs. standard care. Randomization was stratified by usage of oral anticoagulation (OAC) planned to last at least 3 months after surgery. The primary endpoint was a composite of post-operative symptomatic ischemic stroke, transient ischemic attack or imaging findings of silent cerebral ischemic (SCI) lesions.

**Results:**

During a mean follow-up of 3.7 years, 14 (16%) primary events occurred among patients receiving standard surgery vs. 5 (5%) in the group randomized to additional LAACS (hazard ratio 0.3; 95% CI: 0.1–0.8, *p* = 0.02). In per protocol analysis (*n* = 141), 14 (18%) primary events occurred in the control group vs. 4 (6%) in the LAACS group (hazard ratio 0.3; 95% CI: 0.1–1.0, *p* = 0.05).

**Conclusions:**

In a real-world setting, LAACS in addition to elective open-heart surgery was associated with lower risk of post-operative ischemic brain injury. The protective effect was not conditional on AF/OAC status at baseline.

**Trial registration:**

LAACS study, clinicaltrials.gov NCT02378116, March 4th 2015, retrospectively registered.

**Electronic supplementary material:**

The online version of this article (10.1186/s13019-018-0740-7) contains supplementary material, which is available to authorized users.

## Background

Atrial fibrillation (AF) is a common complication after open heart surgery and is associated with both early perioperative and late post-operative stroke [1–5]. Previous studies report incident AF in 10 to 65% of patients after open heart surgery [1, 6], with highest incidences after a combination of coronary artery bypass grafting (CABG) and valve surgery [7]. In patients with non-operative AF, the risk of ischemic stroke is markedly reduced by adequate OAC [8]. However, the management of post-operative AF is still a challenge and is by some regarded as a transient phenomenon not requiring intervention [9, 10]. The risk of bleeding poses a significant limitation to the use of OAC, which consequently increases focus on left atrial appendage (LAA) closure [11, 12], as the LAA is a predilection site for thrombus formation during AF [13, 14]. Importantly, the patient population undergoing open heart surgery is often frail and carry comorbidities such as hypertension and chronic obstructive pulmonary disease, which add risk of future AF and stroke complications [15]. Moreover, a large fraction of these elderly individuals also have subclinical AF that portends an adverse prognosis comparable to recognized AF [16]. It is therefore conceivable that LAACS in conjunction with open heart surgery could mitigate the risk of stroke both in patients with and without overt AF at time of surgery. Notwithstanding, the evidence for routine use of LAACS during elective open-heart surgery is controversial. Accordingly, the primary aim of this study was to investigate the long-term effect of LAACS on cerebral ischemia following scheduled open-heart surgery. We hypothesized that surgical LAACS protects against cerebral ischemia in the following years regardless of AF status at time of surgery.

## Methods

### Study design

From August 2010 to September 2015, we conducted a prospective, randomized, open label study on patients scheduled for open heart surgery to receive either concomitant LAACS or standard care. The LAACS study is registered at clinicaltrials.gov (NCT02378116). The study was initiated at the Department of Thoracic Surgery, University Hospital of Gentofte, Denmark. During 2010, the Department of Thoracic Surgery was transferred and merged with the equivalent department at Rigshospitalet, Copenhagen, where the study continued. Patients were randomized the day before surgery after signing informed consent. To ensure a balanced distribution of patients receiving OAC between the two study-arms, randomization was stratified according to ongoing use of anti-coagulation. This included patients undergoing surgical biological valve replacement until January 2012, as post-operative anti-coagulation was no longer recommended [17].

Patients were randomized 1:1 by a computer-generated randomization in blocks of 16 patients. Since we expected substantial cross-over, procedures were monitored at the end of each operation for cross-over. If one of the study groups reached a discrepancy of 4 between randomization allocation and the actual operation, the randomization block was suspended and substituted by a 3:1 randomization in the next block (n 16) to compensate for the difference.

The study protocol recommended double closure with both purse string and running suture, although this closure method was not mandatory. Patients were invited to pre-surgery magnetic resonance imaging (MRI) scan (MRI-0) whenever possible. Immediately after discharge, all patients were invited for a post-operative baseline brain MRI scan (MRI-1) scheduled between 2 and 4 weeks after discharge and a follow-up MRI (MRI-2) performed at least 6 months after surgery.

### Study population

Consecutive patients undergoing planned first-time open-heart surgery (CABG, valve surgery or a combination of both) during the study period were asked to participate in the trial, provided that their residence was within 40 km radius from the hospital. Major exclusion criteria included endocarditis and implanted pacemaker (see Additional file 1: Table S1 for full inclusion and exclusion criteria). All patients in the LAACS arm were invited to undergo post-operative transesophageal echocardiography (TEE) by a senior cardiologist to visualize the LAA and the quality of the closure. Patients could decline TEE and remain in the study for follow-up.

### MRI analyses

The brain MRI included imaging of cerebrum, cerebellum and brainstem. All MRIs were reviewed by a fellow in radiology with years of experience, and the possibility of consulting unclear cases with a neuro radiologist. Radiologists were blinded to randomization. MRI-criteria for silent cerebral infarcts (SCI) were equivalent to those in the Framingham offspring study, i.e., cavitating lesions ≥3 mm with CSF-like signal intensities on T1, T2 and FLAIR [17]. Both acute and old SCIs were included. Differentiation from dilated Virchow-Robins spaces (dVRS) was attempted using the location criterion (i.e., excluding lesions along perforating or medullary arteries or in the lower third of the basal ganglia) and shape criterion (i.e. oval lesions criteria along the penetrating arteries with intensity close to CSF were considered dVRS) [18].

### Endpoint definitions

The pre-specified endpoint protocol was post-operative cerebral ischemic events defined as a composite of first ischemic stroke or transient ischemic attack (ICD-10: DG450–9), increased amount of SCI between MRI-1 and MRI-2 and post-operative findings of SCI by brain imaging (computed tomography [CT] or MRI) performed in clinical settings unrelated to study enrollment.

Secondary outcomes were strict symptomatic ischemic strokes. That is, ischemic stroke or transient ischemic attack, excluding cerebral ischemic events classified solely on imaging findings and all-cause mortality. We followed all patients for at least 1 year after surgery. The clinical outcomes Ischemic stroke and transient ischemic attack were ascertained by following the patient’s electronic records yearly. Patients vital status were obtained using data from the Central Office of Civil Registration, which comprises all citizens in Denmark. Additionally, telephone interviews were performed after minimum 1 year to ascertain whether the patients had experienced signs of clinically unrecognized cerebral ischemia. If so, it would be registered as a transient ischemic attack.

### Power calculation

We based our power calculations on the incidence of AF among patients undergoing open heart surgery and the expected variation in a combined end-point of clinical stroke, transitory ischemic attack (TIA), and occurrence of silence infarctions, which included findings of newly silent lacunar infarctions with imaging in clinical settings and changes on number of lacunar infarctions from baseline MRI (at discharge) to MR2. Incident stroke occurs in 1–5% of heart surgery patients [19–21], often within the first months following surgery [22]. At the initiation of the study, there were almost no studies with long-term MRI follow-up after open heart surgery [23]. Around 50% of patients undergoing surgery of carotid arteries have lacunar infarctions on MRI [24], and the presence of AF roughly doubles the risk of strokes [25]. We assumed an equivalent prevalence of 50–70% of infarctions counting both subclinical and clinical peri-operative strokes/TIA. This is equivalent to findings on CABG surgery [26] and valve surgery [23], respectively, where MRI changes occur in 40–70% of patients. Therefore, we assumed that closure of the LAA would reduce findings on MRI scans and clinical strokes/TIA from a total of 60 to 35% with a 5% margin. With a significance level of 5%, we calculated that we could demonstrate this benefit with a 5% margin and 90% power by including 90 patients. After the initiation of the study, it became apparent that MRI scans were not feasible for many patients. Hence, we recalculated the number of patients needed based on newer data on occurrence of stroke after heart surgery [27–30], considering a three- to five-fold increased risk of stroke in patients with non-contracting, patent LAA who had undergone MAZE-procedure during surgery [31] combined with an occurrence of images of infarction after CABG over 20% [32], the latter including MRI changes [23, 26]. Without other available evidence on truly long-term follow-up studies after heart surgery associated to AF, we calculated that we could investigate the protective effect of LAACS based on any signs of cerebral infarction (stroke, TIA or imaging evidence of silent cerebral ischemia) through randomization of 400 patients, if LAACS could reduce events from 65 to 45% with a significance level of 5 and 91% power. With 88% power, a second calculation showed a necessity of including 100 patients in each group. Therefore, we maintained our plan to randomize 200 patients.

### Statistical analysis

All analyses were performed using SAS statistical software package version 9.4 (SAS Institute Inc., Cary, NC). Continuous variables are presented as mean±standard deviation and categorical variables as number and percentages. After inspection for normality, differences between the LAACS and control group were evaluated by Student’s t-test (continuous variables) and by Chi-square and Fisher’s exact test (categorical variables) where appropriate. Cause specific Cox time to event analysis was used to estimate the rate ratios for incident stroke and all-cause mortality according to randomization. A cumulative probability plot of incident stroke according to randomization was generated by Fine and Gray competing risk regression using death as a competing event [33]. All outcome analyses were performed as intention to treat and per protocol. Patients were considered to have complied with the treatment protocol when LAAC was performed according to randomization. A sensitivity analysis was performed to evaluate model dependence on imaging findings by comparing overall results with a model that only included events due to symptomatic ischemic stroke or transient ischemic attack. A two-tailed *p*-value < 0.05 was regarded as statistically significant.

### Ethics

The project was approved by Regional Ethics Committee of Capital Region Denmark (protocol number H-3-2010-017) and follows the Helsinki declaration.

## Results

Of 914 patients invited to participate, 205 (22.4%) were enrolled. Of those, 187 (91.2%) were randomized and 141 (75.4%) ultimately followed the treatment protocol (see Additional file 2: Figure S1). During the second year of the study, discrepancy between randomization and performed procedure reached a difference of four (due to overweight of patients randomized to LAACS who did not undergo closure), and the following randomization blocks of 16 patients were switched to 3:1 randomization. The rest of the study randomization continued 1:1.

Two patients randomized to additional LAACS crossed over due to technical difficulties during the operation, and the LAACS procedure was avoided. In five patients with known paroxysms of AF, the surgeons changed the planned operation adding intra-operative ablation and closing the left appendage, despite randomization to the control group. Baseline characteristics of randomized participants are showed in Table 1. No adverse events such as bleeding due to LAACS procedure were recorded. 75 (40%) patients underwent both of the planned brain MRIs. Thirty patients had brain infarctions in pre-surgery MRI. Of 74 patients who underwent subsequent MRI shortly after discharge, 11 had SCI at post-discharge MRI. Among 75 available sets of post-discharge and long-term MRI, only two had new SCI, one in each group.

**Table 1:** Baseline characteristics according to randomized left atrial appendage closure

Variable	Not closed (*n* = 86)	Closed (*n* = 101)	
Age – years	69.3 ± 8.8	67.6 ± 9.6	
Men - *n* (%)	75 (87.2)	84 (83.2)	
Clinical characteristics			
Congestive heart failure - *n* (%)	15 (17.9)	16 (15.8)	
Atrial fibrillation - *n* (%)	12 (12.8)	18 (16.8)	
Diabetes - *n* (%)	19 (22.1)	31 (30.7)	
Hypertension - *n* (%)	60 (69.8)	75 (74.3)	
CHADS-VASc – unit	2.9 ± 1.4	2.9 ± 1.5	
Prior stroke - *n* (%)	15 (17.4)	11 (10.9)	
Chronic kidney disease^a^ - n (%)	14 (16.3)	15 (14.9)	
Medicine			
ASA - *n* (%)	69 (80.2)	75 (74.3)	
Clopidogrel - *n* (%)	14 (16.3)	19 (18.8)	
OAC			
*VKA* - *n* (%)	26 (30.2)	36 (35.6)	
*NOAC* - *n* (%)	2 (2.2)	2 (2.0)	
Beta-blocker - *n* (%)	47 (54.7)	61 (60.4)	
Verapamil - *n* (%)	4 (4.5)	2 (2.0)	
Calcium-blocker - *n* (%)	19 (21.3)	34 (33.7)	
Digoxin - *n* (%)	5 (5.6)	3 (3.0)	
Renin-angiotensin system blocker - n (%)	40 (46.5)	54 (53.5)	
Amiodarone - n (%)	23 (26.7)	18 (17.8)	
Statin - n (%)	74 (86.0)	81 (80.2)	
Procedural characteristics			
Surgery type			
*AVR only* - n (%)	17 (19.1)	17 (16.8)	
*AVR with CABG* - n (%)	20 (22.5)	22 (21.8)	
*AVR with aortic surgery* - n (%)	1 (1.1)	1 (1.0)	
*AVR with MVR* - n (%)	0 (0)	2 (2.0)	
*Aortic surgery only* - n (%)	1 (1.1)	0 (0)	
*CABG only* - n (%)	40 (46.5)	50 (49.5)	
*CABG with MVR* - n (%)	2 (2.3)	2 (2.0)	
*MVR only* - n (%)	4 (4.5)	7 (6.9)	
*Tricuspid surgery only* - n (%)	1 (1.2)	0 (0)	
Perioperative atrial fibrillation - n (%)	52 (60.5)	50 (50.0)	

*Abbreviations CHADS-VASc* Congestive heart failure, hypertension, age [≥75 years], diabetes, stroke – peripheral vascular disease, age [≥65 years], sex-category, *OAC* Oral anticoagulation, *VKA* Vitamin K-antagonist, *NOAC* Novel oral anticoagulation, IQR: interquartile range

^a^eGFR< 30 ml/min

There were no detectable differences in randomization to LAACS, age or gender between patients that had the planned brain MRI scans vs. those that did not (all *p* > 0.35, Additional file 3: Table S2). 10 patients from the LAACS group accepted the invitation to undergo TEE (mean 524 days after surgery date). In none of the cases could the lumen of the LAA or any flow from the appendage orifice be identified.

### Clinical outcomes

**Fig. 1:**
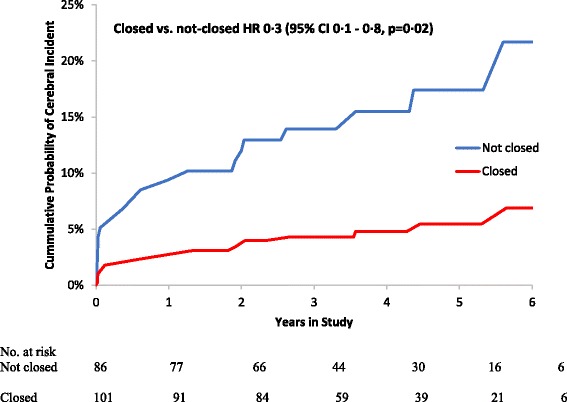
Cumulative probability of primary events (combined ischemic stroke, transitory ischemic stroke or silent ischemic images) according to randomized LAACS (left atrial appendage closure with surgery)

**Fig. 2:**
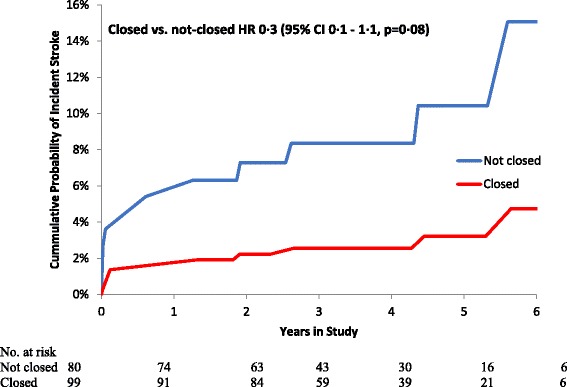
Cumulative probability of symptomatic ischemic stroke or transient ischemic attack (image findings excluded) according to randomized LAACS (left atrial appendage closure with surgery)

Follow up was up to 6 years, mean 3.7 ± 1.6 years (totaling 684 patient years of follow-up). End of study was 1 year after reaching our pre-specified sample size of 200 patients. Five patients were lost to follow-up for the primary endpoint and 24 (13%) died**.** At end of study telephone-interviews, no patient was suspected to have suffered a clinically unrecognized ischemic cerebral event. In the intention-to-treat analysis, there were 5 (5%) primary events in the LAACS-group and 14 (16%) in the control group (hazard ratio 0.3; 95% CI: 0.1–0.8, *p* = 0.02, Table 2, Fig. 1 and Additional file 4: Table S3 Contingency table of individual events). Tests of interaction revealed no dependency of the preventative effect of LAACS on baseline AF status, CHA_2_DS_2_-VASc score or use of OAC (*p* = 0.55, *p* = 0.56 and *p* = 0.49 for interaction, respectively). Secondary outcome analysis revealed no detectable difference in all-cause mortality (*p* = 0.79) between the LAACS group (*n* = 12, 12%) vs. the controls (*n* = 12, 14%, Table 2). Similarly, in a sensitivity analysis that excluded the eight patients that underwent primary events only based on radiological findings, there was a trend but no longer a significant reduction in ischemic strokes among patients randomized to LAACS (*p* = 0.08, Table 2 and Fig. 2).

**Table 2:** Proportion of patients meeting endpoints according to randomized left atrial appendage closure, stratified by use of anti-coagulants

Endpoint	Not closed (*n* = 86)	Closed (*n* = 101)	Hazard ratio	*P*-value
Primary events^a^	14 (16.3%)	5 (5.0%)	0.3 [95% CI: 0.1–0.8]	0.0168
Clinical stroke^b^	8 (10.0%)	3 (3.0)	0.3 [95% CI: 0.1–1.1]	0.0763
Death	12 (14.0%)	12 (11.9%)	0.8 [95% CI: 0.43–1.9]	0.6562

^a^Defined as first of postoperative symptomatic ischemic stroke, transient ischemic attack or imaging evidence of new silent infarct

^b^Excluding 8 patients that were classified as primary events due to imaging findings

In the per protocol sensitivity analysis (Table 3), there were 4 (6%) primary events in the LAACS group vs. 14 (18%) in the control arm (hazard ratio 0.3; 95% CI: 0.1–1.0, *p* = 0.05). Of note, in the control group, 9 (64%) of the 14 primary events occurred beyond the first year of follow-up (Fig. 1). Secondary outcome analyses again revealed no detectable difference in overall survival or strict symptomatic ischemic strokes between the two study-arms (Table 3).

**Table 3:** Proportion of patients meeting endpoints according to per-protocol left atrial appendage closure stratified by use of anti-coagulants

Endpoint	Not closed (*n* = 77)	Closed (*n* = 64)	Hazard ratio	*P*-value
Primary events^a^	14 (18.2%)	4 (6.3%)	0.3 [95% CI: 0.1–0.9]	0.0237
Clinical stroke^b^	8 (11.3%)	2 (3.2%)	0.3 [95% CI: 0.1–1.3]	0.0907
Death	10 (13.0%)	6 (9.4%)	0.7 [95% CI: 0.3–2.3]	0.7067

^a^Defined as first of postoperative symptomatic ischemic stroke, transient ischemic attack or imaging evidence of new silent infarct

^b^Excluding 8 patients that were classified as primary events due to imaging findings

The datasets used during the current study are available from the corresponding author on reasonable request.

## Discussion

This is the first randomized study where LAACS in addition to first-time open-heart surgery (CABG, valve or combination of both) seems to protect against post-operative cerebral ischemic events several years after surgery. Despite a substantial number of cross-overs, results were consistent in the intention-to-treat and per-protocol analyses. Furthermore, the impact during long-term follow-up should become visible in patients with AF, which may be offset with effective anticoagulation.

Thus, LAACS seems as a safe, low cost and easily feasible procedure that may mitigate risk of post-operative cerebral ischemia among patients undergoing planned heart surgery.

Although encouraging, these results must be interpreted cautiously. The study is not powered to demonstrate a protection against stroke. Furthermore, the rationale for including SCI in the composite primary endpoint is rather indirect [34, 35]. Yet, we found a trend towards higher stroke and TIA events in the control group (*p* = 0.07), which seems to support the relevance of SCI as true brain damage.

Comparing our findings to available literature, it is generally accepted that there is very little evidence on the effects of surgical closure of the LAA [36]. A previous study from 2000 by Johnson et al. showed no safety issues regarding LAACS among the 437 patients included, where of which 43 appendages were stapled and 391 were sutured. [37] In a recent systematic review of LAA closure, the authors concluded that there are no randomized clinical trials and that published evidence is insufficient to assess the benefits of LAACS but there is seemingly no adverse risk associated with the procedure [36].

Of note, most of the primary events in the present control group were late ischemic strokes well clear of the time of surgery and the complex array of risk factors related to perioperative stroke. This is in support of the theory that post-operative ischemic events by large are unrelated to the surgical procedure per se, but instead originate from LAA thrombus formation induced by AF. The facts that LAA is a prevalent site of thrombus formation during AF and that the incidence of AF after open heart surgery is as high as 50–60% further supports this hypothesis [38]. In turn, this may also explain why the effect of LAACS was not altered by baseline AF status, as there is a general consensus that post-operative AF is associated with late post-operative stroke [1, 22, 39]. Thus, LAACS may act by blocking a causal mechanism for late ischemic strokes induced by either pre- or post-operative AF.

A major concern of surgical versus percutaneous LAA closure is the risk of incomplete closure [40], although there is very little evidence that supports this notion [11]. In our study, we found no signs of incomplete LAACS among the 10 participants that underwent TEE. With any of the surgical techniques, there is a risk of incomplete LAACS, though it varies greatly depending upon the surgical technique utilized and the definition of surgical failure [41]. Nevertheless, a recent experimental study in vitro comparing epicardial closure supports double closure with both purse string and running suture as recommended in our protocol [42].

### Study limitations

The study was halted before reaching the planned randomization of 200 patients due to competing studies recruiting patients for heart surgery. Nevertheless, according to our findings, it would be sufficient to include 35 patients in each group with 90% power to find a difference on the combination of stroke, TIA and SCI 2 years after surgery with a significance level of 5%. Furthermore, due to the frailty of the patients included, the discomfort of cerebral MRI and the physically- and mentally challenging period postoperatively, it was only possible to perform full sets of MRI scans in 75 patients. However, the patients who were not able to participate in all MRI scans agreed to stay active in the study for future clinical follow up. The fact that all patients did not undergo the planned MRI scans may have introduced a selection-bias, as frail patients may be more prone to decline the brain MRI scans. However, results from sensitivity analyses restricted to symptomatic ischemic strokes support the results that included imaging findings. Another limitation is the fact that only 10 patients accepted postoperative TEE. An unnoticed incomplete LAA closure, may thus have provoked events.

## Conclusion

In a real-world setting, LAACS during elective open-heart surgery was associated with a lower risk of ischemic cerebral events following open heart surgery.

## Additional files

Additional file 1: **Table S1.** Complete inclusion/exclusion criteria. Table listing inclusion and exclusion criteria. (DOCX 13 kb)

Additional file 2: **Figure S1**. Flow-chart. Figure showing the flow-chart from screening until randomization (DOCX 50 kb)

Additional file 3: **Table S2**. Baseline characteristics among patients that underwent planned brain MRI scans vs. those that did not. Table showing characteristics of patients who underwent MRI compared to those who did not. (DOCX 19 kb)

Additional file 4: **Table S3**. Breakdown of primary events according to randomized treatment. Table showing brake-down of events in patients with closed LAA compared with the control group where LAA remained open. (DOCX 19 kb)

## Abbreviations

AF: Atrial fibrillation
CABG: Coronary artery bypass grafting
CI: Confidence interval
CT: Computed tomography
dVRS: Dilated Virchow-Robins spaces
eGFR: Estimated glomerular filtration rate
HR: Hazard ratio
ICD: International classification of diseases
IQR: Inter-quartile range
LAA: Left atrial appendage
LAACS: Left atrium appendage closure with surgery
MRI: Magnetic resonance imaging
NOAC: Novel oral anticoagulation
OAC: Oral anti-coagulants
SCI: Silent cerebral infarcts
TEE: Transesophageal echocardiography
TIA: Transitory ischemic attack (TIA)
VKA: Vitamin K-antagonist

## References

[1] McDonagh DL, Berger M, Mathew JP, Graffagnino C, Milano CA, Newman MF (2014). Neurological complications of cardiac surgery. Lancet Neurol.

[2] Murdock DK, Rengel LR, Schlund A, Olson KJ, Kaliebe JW, Johnkoski JA, Riveron FA (2003). Stroke and atrial fibrillation following cardiac surgery. WMJ.

[3] Lubitz SA, Yin X, Rienstra M, Schnabel RB, Walkey AJ, Magnani JW, Rahman F, McManus DD, Tadros TM, Levy D (2015). Long-term outcomes of secondary atrial fibrillation in the community: the Framingham heart study. Circulation.

[4] Saxena A, Dinh D, Dimitriou J, Reid C, Smith J, Shardey G, Newcomb A (2013). Preoperative atrial fibrillation is an independent risk factor for mid-term mortality after concomitant aortic valve replacement and coronary artery bypass graft surgery. Interact Cardiovasc Thorac Surg.

[5] Ahlsson A, Fengsrud E, Bodin L, Englund A (2010). Postoperative atrial fibrillation in patients undergoing aortocoronary bypass surgery carries an eightfold risk of future atrial fibrillation and a doubled cardiovascular mortality. Eur J Cardiothorac Surg.

[6] Maisel WH. Left atrial appendage occlusion -- closure or just the beginning? N Engl J Med. 2009;360(25):2601-3. 10.1056/NEJMp0903763 19474420

[7] Maesen B, Nijs J, Maessen J, Allessie M, Schotten U (2012). Post-operative atrial fibrillation: a maze of mechanisms. Europace.

[8] Aguilar MI, Hart R (2005). Oral anticoagulants for preventing stroke in patients with non-valvular atrial fibrillation and no previous history of stroke or transient ischemic attacks. Cochrane Database Syst Rev.

[9] Frendl G, Sodickson AC, Chung MK, Waldo AL, Gersh BJ, Tisdale JE, Calkins H, Aranki S, Kaneko T, Cassivi S (2014). 2014 AATS guidelines for the prevention and management of perioperative atrial fibrillation and flutter for thoracic surgical procedures. J Thorac Cardiovasc Surg.

[10] Kowey PR, Stebbins D, Igidbashian L, Goldman SM, Sutter FP, Rials SJ, Marinchak RA (2001). Clinical outcome of patients who develop PAF after CABG surgery. Pacing Clin Electrophysiol.

[11] Garcia-Fernandez MA, Perez-David E, Quiles J, Peralta J, Garcia-Rojas I, Bermejo J, Moreno M, Silva J (2003). Role of left atrial appendage obliteration in stroke reduction in patients with mitral valve prosthesis: a transesophageal echocardiographic study. J Am Coll Cardiol.

[12] Reddy VY, Doshi SK, Sievert H, Buchbinder M, Neuzil P, Huber K, Halperin JL, Holmes D, Investigators PA (2013). Percutaneous left atrial appendage closure for stroke prophylaxis in patients with atrial fibrillation: 2.3-year follow-up of the PROTECT AF (watchman left atrial appendage system for embolic protection in patients with atrial fibrillation) trial. Circulation.

[13] Freedman B, Potpara TS, Lip GY (2016). Stroke prevention in atrial fibrillation. Lancet.

[14] Lin AC, Knight BP (2015). Left atrial appendage closure. Prog Cardiovasc Dis.

[15] Mathew JP, Fontes ML, Tudor IC, Ramsay J, Duke P, Mazer CD, Barash PG, Hsu PH, Mangano DT (2004). A multicenter risk index for atrial fibrillation after cardiac surgery. JAMA.

[16] Sanna T, Diener HC, Passman RS, Di Lazzaro V, Bernstein RA, Morillo CA, Rymer MM, Thijs V, Rogers T, Beckers F (2014). Cryptogenic stroke and underlying atrial fibrillation. N Engl J Med.

[17] Das RR, Seshadri S, Beiser AS, Kelly-Hayes M, Au R, Himali JJ, Kase CS, Benjamin EJ, Polak JF, O'Donnell CJ (2008). Prevalence and correlates of silent cerebral infarcts in the Framingham offspring study. Stroke.

[18] Giele JL, Witkamp TD, Mali WP, van der Graaf Y, Group SS (2004). Silent brain infarcts in patients with manifest vascular disease. Stroke.

[19] Lahtinen J, Biancari F, Salmela E, Mosorin M, Satta J, Rainio P, Rimpilainen J, Lepojarvi M, Juvonen T (2004). Postoperative atrial fibrillation is a major cause of stroke after on-pump coronary artery bypass surgery. Ann Thorac Surg.

[20] Blackshear JL, Odell JA (1996). Appendage obliteration to reduce stroke in cardiac surgical patients with atrial fibrillation. Ann Thorac Surg.

[21] Salazar JD, Wityk RJ, Grega MA, Borowicz LM, Doty JR, Petrofski JA, Baumgartner WA (2001). Stroke after cardiac surgery: short- and long-term outcomes. Ann Thorac Surg.

[22] Kollar A, Lick SD, Vasquez KN, Conti VR (2006). Relationship of atrial fibrillation and stroke after coronary artery bypass graft surgery: when is anticoagulation indicated?. Ann Thorac Surg.

[23] Knipp SC, Matatko N, Schlamann M, Wilhelm H, Thielmann M, Forsting M, Diener HC, Jakob H (2005). Small ischemic brain lesions after cardiac valve replacement detected by diffusion-weighted magnetic resonance imaging: relation to neurocognitive function. Eur J Cardiothorac Surg.

[24] Wilson DA, Mocco J, D'Ambrosio AL, Komotar RJ, Zurica J, Kellner CP, Hahn DK, Connolly ES, Liu X, Imielinska C (2008). Post-carotid endarterectomy neurocognitive decline is associated with cerebral blood flow asymmetry on post-operative magnetic resonance perfusion brain scans. Neurol Res.

[25] Harthun NL, Stukenborg GJ (2010). Atrial fibrillation is associated with increased risk of perioperative stroke and death from carotid endarterectomy. J Vasc Surg.

[26] Floyd TF, Shah PN, Price CC, Harris F, Ratcliffe SJ, Acker MA, Bavaria JE, Rahmouni H, Kuersten B, Wiegers S (2006). Clinically silent cerebral ischemic events after cardiac surgery: their incidence, regional vascular occurrence, and procedural dependence. Ann Thorac Surg.

[27] Garg S, Sarno G, Gutierrez-Chico JL, Garcia-Garcia HM, Gomez-Lara J, Serruys PW, investigators A-I (2011). Five-year outcomes of percutaneous coronary intervention compared to bypass surgery in patients with multivessel disease involving the proximal left anterior descending artery: an ARTS-II sub-study. EuroIntervention.

[28] Hedberg M, Boivie P, Engstrom KG (2011). Early and delayed stroke after coronary surgery - an analysis of risk factors and the impact on short- and long-term survival. Eur J Cardiothorac Surg.

[29] Bucerius J, Gummert JF, Borger MA, Walther T, Doll N, Onnasch JF, Metz S, Falk V, Mohr FW (2003). Stroke after cardiac surgery: a risk factor analysis of 16,184 consecutive adult patients. Ann Thorac Surg.

[30] Korn-Lubetzki I, Oren A, Asher E, Dano M, Bitran D, Fink D, Steiner-Birmanns B (2007). Strokes after cardiac surgery: mostly right hemispheric ischemic with mild residual damage. J Neurol.

[31] Buber J, Luria D, Sternik L, Raanani E, Feinberg MS, Goldenberg I, Nof E, Gurevitz O, Eldar M, Glikson M (2011). Left atrial contractile function following a successful modified maze procedure at surgery and the risk for subsequent thromboembolic stroke. J Am Coll Cardiol.

[32] Gottesman RF, Sherman PM, Grega MA, Yousem DM, Borowicz LM, Selnes OA, Baumgartner WA, McKhann GM (2006). Watershed strokes after cardiac surgery: diagnosis, etiology, and outcome. Stroke.

[33] Jason P, Fine RJG (1999). A Proportional Hazards Model for the subdistribution of a competing risk. J Am Stat Assoc.

[34] Gaita F, Corsinovi L, Anselmino M, Raimondo C, Pianelli M, Toso E, Bergamasco L, Boffano C, Valentini MC, Cesarani F (2013). Prevalence of silent cerebral ischemia in paroxysmal and persistent atrial fibrillation and correlation with cognitive function. J Am Coll Cardiol.

[35] Stefansdottir H, Arnar DO, Aspelund T, Sigurdsson S, Jonsdottir MK, Hjaltason H, Launer LJ, Gudnason V (2013). Atrial fibrillation is associated with reduced brain volume and cognitive function independent of cerebral infarcts. Stroke.

[36] Noelck N, Papak J, Freeman M, Paynter R, Low A, Motu'apuaka M, Kondo K, Kansagara D (2016). Effectiveness of left atrial appendage exclusion procedures to reduce the risk of stroke: a systematic review of the evidence. Circ Cardiovasc Qual outcomes.

[37] Johnson WD, Ganjoo AK, Stone CD, Srivyas RC, Howard M (2000). The left atrial appendage: our most lethal human attachment! Surgical implications. Eur J Cardiothorac Surg.

[38] Patel D, Gillinov MA, Natale A (2008). Atrial fibrillation after cardiac surgery: where are we now?. Indian Pacing Electrophysiol J.

[39] Creswell LL, Schuessler RB, Rosenbloom M, Cox JL (1993). Hazards of postoperative atrial arrhythmias. Ann Thorac Surg.

[40] Aryana A, d'Avila A (2016). Incomplete closure of the left atrial appendage: implication and management. Curr Cardiol Rep.

[41] Katz ES, Tsiamtsiouris T, Applebaum RM, Schwartzbard A, Tunick PA, Kronzon I (2000). Surgical left atrial appendage ligation is frequently incomplete: a transesophageal echocardiograhic study. J Am Coll Cardiol.

[42] Mirow N, Vogt S, Irqsusi M, Moosdorf R, Kirschbaum A (2017). Epicardial left atrial appendage closure-comparison of surgical techniques in an ex vivo model. J Thorac Dis.

