# Supplementary material for: Adding left atrial appendage closure to open heart surgery provides protection from ischemic brain injury six years after surgery independently of atrial fibrillation history: the LAACS randomized study
Source: J Cardiothorac Surg. 2018 May 23;13:53. doi: 10.1186/s13019-018-0740-7 (PMC5967101; doi:10.1186/s13019-018-0740-7)
Supplement: Supplementary file 1 — Table S1. Complete inclusion/exclusion criteria. Table listing inclusion and exclusion criteria. (DOCX 13 kb) [file 13019_2018_740_MOESM1_ESM.docx]

**Table S1** Summary of inclusion and exclusion criteria

| **Inclusion Criteria** | **Exclusion Criteria** |
| --- | --- |
| Age > 18 years | Off-pump surgery |
| Planned CABG | Endocarditis |
| Planned valve surgery alone or combined with CABG | Patients with Pacemaker or implanted electronic medical devices or other metal implants not MRI safe |
| Signed informed consent. | Planned pacemaker after surgery |
|  | Residence more than 40 Km away from the hospital |
