# Supplementary material for: Adding left atrial appendage closure to open heart surgery provides protection from ischemic brain injury six years after surgery independently of atrial fibrillation history: the LAACS randomized study
Source: J Cardiothorac Surg. 2018 May 23;13:53. doi: 10.1186/s13019-018-0740-7 (PMC5967101; doi:10.1186/s13019-018-0740-7)
Supplement: Supplementary file 2 — Figure S1. Flow-chart. Figure showing the flow-chart from screening until randomization (DOCX 50 kb) [file 13019_2018_740_MOESM2_ESM.docx]

**Figure S2** Flow-Chart

**LAACS Flow Diagram**

## Screenning

Lost to follow-up (n= 1)

Randomized (n=187)

## Follow-Up

Analysed (n= 86)

Analysed (n=101)

Lost to follow-up (n= 1)

## Enrollment

Allocated to control (n=86)

♦ Received allocated intervention (n=77)

♦ Did not receive allocated intervention (n= 9)

## Allocation

Allocated to LAAC (n=101)

♦ Received allocated intervention (n=64)

♦ Did not receive allocated intervention (n= 37)

Screened > 6000 patients

Of

Invited eligible (n=915)

Enrolled (n = 205)

## Analysis
