# Supplementary material for: Adding left atrial appendage closure to open heart surgery provides protection from ischemic brain injury six years after surgery independently of atrial fibrillation history: the LAACS randomized study
Source: J Cardiothorac Surg. 2018 May 23;13:53. doi: 10.1186/s13019-018-0740-7 (PMC5967101; doi:10.1186/s13019-018-0740-7)
Supplement: Supplementary file 3 — Table S2. Baseline characteristics among patients that underwent planned brain MRI scans vs. those that did not. Table showing characteristics of patients who underwent MRI compared to those who did not. (DOCX 19 kb) [file 13019_2018_740_MOESM4_ESM.docx]

**Additional file 4 – Table S4:** Breakdown of primary events according to randomized treatment.

| Components of primary events | Randomization* | | |
| --- | --- | --- | --- |
|  | Not closed N=86 | Closed N=101 | Total N=187 |
| No signs cerebral ischemia | \| N=72 \| \| --- \| \| 83·7% \| | \| N=96 \| \| --- \| \| 95·1% \| | \|  \| \| --- \| \| N = 168  89·8% \| \|  \| \|  \| |
| Symptomatic ischemic stroke | \| N=6 \| \| --- \| \| 7·0% \| | \| N=2 \| \| --- \| \| 2·0% \| | \|  \| \| --- \| \| N=8 \| \| 4·3% \| \|  \| |
| Transient ischemic attack | \| N=2 \| \| --- \| \| 2·3% \| | \| N=1 \| \| --- \| \| 1·00% \| | \| N=3 \| \| --- \| \| 1·6% \| \|  \| \|  \| |
| Findings of infarction by imaging in clinical setting | \| N=4 \| \| --- \| \| 4·7% \| | \| N=1 \| \| --- \| \| 1·0% \| | \| N=5 \| \| --- \| \| 2·7% \| \|  \| \|  \| |
| Findings of new SCI on planned brain MRI | \| N=2 \| \| --- \| \| 2·3% \| | \| N=1 \| \| --- \| \| 1·0% \| | \| N=3 \| \| --- \| \| 1·6% \| \|  \| \|  \| |

Abbreviations: SCI = silent cerebral infarction.

* Left atrial appendage closure vs. standard care
