# Supplementary material for: Adding left atrial appendage closure to open heart surgery provides protection from ischemic brain injury six years after surgery independently of atrial fibrillation history: the LAACS randomized study
Source: J Cardiothorac Surg. 2018 May 23;13:53. doi: 10.1186/s13019-018-0740-7 (PMC5967101; doi:10.1186/s13019-018-0740-7)
Supplement: Supplementary file 4 — Table S3. Breakdown of primary events according to randomized treatment. Table showing brake-down of events in patients with closed LAA compared with the control group where LAA remained open. (DOCX 19 kb) [file 13019_2018_740_MOESM3_ESM.docx]

**Additional file 3 – Table S3:** Baseline characteristics among patients that underwent planned brain MRI scans vs. those that did not.

| **Variable** | **-brain MRI**  (n=112) | **+brain MRI**  (n=75) | P-value |
| --- | --- | --- | --- |
| Age – years | 68·6±9·0 | 68·2±9·7 | 0·75 |
| Men - (%) | 83·0% | 88·0% | 0·35 |
| **Clinical characteristics** |  |  |  |
| Congestive heart failure - (%) | 18·4% | 14·9% | 0·54 |
| Atrial fibrillation - (%) | 13·8% | 17·3% | 0·51 |
| Diabetes - (%) | 29·1% | 23·4% | 0·39 |
| Hypertension - (%) | 66·4% | 81·1% | 0·03 |
| CHADS-VASc – unit | 2·9±1·5 | 3·0±1·4 | 0·46 |
| Prior stroke - (%) | 13·8% | 14·9% | 0·83 |
| Chronic kidney disease* - (%) | 15·4% | 15·5% | 0·98 |
| **Medicine** |  |  |  |
| ASA - (%) | 75·7% | 79·7% | 0·52 |
| Clopidogrel - (%) | 17·9% | 16·4% | 0·80 |
| OAC |  |  |  |
| *VKA* - (%) | 34·3% | 34·4% | 1·00 |
| *NOAC* - (%) | 2·8% | 4·2% | 1·00 |
| Beta-blocker - (%) | 57·6% | 58·1% | 0·94 |
| Verapamil - (%) | 2·8% | 4·1% | 0·69 |
| Calcium-blocker - (%) | 31·1% | 24·3% | 0·32 |
| Digoxin - (%) | 3·8% | 5·5% | 0·59 |
| Renin-angiotensin system blocker - (%) | 52·8% | 46·0% | 0·36 |
| Amiodarone - (%) | 21·7% | 21·4% | 0·97 |
| Statin - (%) | 81·3% | 85·1% | 0·50 |
| **Procedural characteristics** |  |  |  |
| Left atrial appendage closure – (%) | 54·5% | 53·3% | 0·88 |
| Surgery type |  |  |  |
| *AVR only* - (%) | 22·3% | 12·0% | 0·07 |
| *AVR with CABG* - (%) | 22·3% | 21·3% | 0·87 |
| *AVR with aortic surgery* - (%) | 1·8% | 0% | 0·52 |
| *AVR with MVR* - (%) | 0% | 2·7% | 0·16 |
| *Aortic surgery only* - (%) | 0% | 1·3% | 0·40 |
| *CABG only* - (%) | 42·0% | 57·3% | 0·04 |
| *CABG with MVR* - (%) | 0·9% | 4·0% | 0·30 |
| *MVR only* - (%) | 8·9% | 1·3% | 0·05 |
| *Tricuspid surgery only* - (%) | 0·9% | 0% | 0·41 |
| Perioperative atrial fibrillation - (%) | 57·7% | 50·7% | 0·35 |

Abbreviations - CHADS-VASc: Congestive heart failure, hypertension, age [≥75 years], diabetes, stroke – peripheral vascular disease, age [≥65 years], sex-category, OAC: Oral anticoagulation, VKA: Vitamin K-antagonist, NOAC: Novel oral anticoagulation, IQR: interquartile range.

*eGFR<30ml/min
